# Reduction of H3K27cr Modification During DNA Damage in Colon Cancer

**DOI:** 10.3389/fonc.2022.924061

**Published:** 2022-07-22

**Authors:** Meijian Liao, Weiwei Chu, Xiaolin Sun, Wendan Zheng, Shoucui Gao, Danhua Li, Dongsheng Pei

**Affiliations:** ^1^ Department of Pathology, Xuzhou Medical University, Xuzhou, China; ^2^ School of Pharmaceutical Sciences (Shenzhen), Sun Yat-sen University, Shenzhen, China

**Keywords:** histone crotonylation, H3K27cr, DNA damage, SIRT6, etoposide

## Abstract

DNA damage plays an essential role in the initiation and development of colon cancer. Histone crotonylation is a newly discovered post-translational modification that is thought to promote gene expression. Whether histone crotonylation plays a role in DNA damage of cancer remains unknown, as does the putative underlying molecular mechanism. This study aimed to investigate the relationship between histone crotonylation and DNA damage of colon cancer using multiple bioinformatics analysis and western blotting. We discovered that genes with promoter occupied by histone crotonylation were associated with the activity of DNA damage in colon cancer patients. Additionally, we uncovered that the level of crotonylation on Lys27 of histone H3 (H3K27cr) decreased during camptothecin and etoposide treatment. Interestingly, sirtuin 6 was found to regulate the cellular level of H3K27cr. Taking these data together, our study provided a new perspective about histone crotonylation and DNA damage in colon cancer.

## Introduction

A large number of chemicals, physical stimuli, and biological processes, such as reactive oxygen species, UV and ionizing radiation, DNA replication, and gene transcription, can generate DNA lesions and impair genomic integrity. Recent evidence demonstrate that DNA damage causes genomic mutations and epimutations, suggesting that DNA damage accumulation may trigger cancer and enhance its development. Colon cancer is the third most common cancer worldwide. DNA damage and DNA repair play a critical role in the initiation and development of colon cancer. Colon cancer cells with DNA damage accumulation may trigger cell cycle arrest (1) or apoptosis (2). The signature based on genes related to DNA damage and DNA repair can serve as a marker of colon cancer prognosis (3). In addition, DNA damage can also serve as a predictor of prognosis in colorectal liver metastases (4).

Lysine crotonylation (Kcr) is a newly identified post-translational modification (5). The Kcr of total protein is found to participate in the proliferation, invasion, and migration of HeLa cells (6). The Kcr of non-histone is believed to play a critical role in tumorigenesis (7). Histone crotonylation is overexpressed in prostate cancer tissues (8). Besides this, the Kcr of total protein is upregulated in colon cancer (9). However, the detailed molecular mechanisms of regulating histone crotonylation have not been completely understood until now. Only a small number of writers, readers, and erasers are discovered. The sirtuin family of deacetylases (SIRT1/2/3) and class I histone deacetylases (HDAC1/2/3) have been discovered to contain decrotonylase activity (10, 11). The sirtuin family is the first histone decrotonylases identified, which recognizes crotonyl lysine *via* π−π stacking interaction.

A number of evidence reveal that gene expression is turned off at the DNA damage sites (12), and histone crotonylation is thought to facilitate gene transcription. Therefore, we considered that histone crotonylation may be involved in DNA damage. Actually, the Kcr of RPA1 protein has been demonstrated in mediating the repair of camptothecin (CPT)-induced DNA damage (13). Additionally, the level of H3K9cr is decreased during DNA damage response (14). However, in cancer patients, whether histone crotonylation is also associated with DNA damage remains unclear. In this study, we aimed to explore the association between histone crotonylation and DNA damage in colon cancer. We discovered that genes with promoter occupied by histone crotonylation were enriched in colon cancer patients with a low activity of DNA damage. In colon cancer patients, the expression of most genes with promoter occupied by H3K27cr and the activity of DNA damage were negatively correlated.

Previous studies have shown that SIRT6, a member of sirtuin family, rapidly recognizes and accumulates at the DNA break sites and promotes chromatin relaxation and DNA repair (15). Since histone crotonylation is related to DNA damage, we considered that there might be a link between SIRT6 and histone crotonylation. Therefore, whether SIRT6 altered the level of histone crotonylation was also investigated. We discovered that genes with promoter occupied by histone crotonylation were enriched in colon cancer patients with a low activity of SIRT6. Additionally, inhibition of SIRT6 increased the level of H3K27cr in HCT-116 cells.

## Materials and Methods

### Information of Datasets

The UCSC Xena database (https://xena.ucsc.edu) is an online exploration tool for visualization of gene expression profiles and protein activities (16). The PARADIGM-inferred pathway levels (paradigm IPLs) are always used to infer protein activities based on gene expression and copy number data (17). Complexes or abstract processes of paradigm IPLs in The Cancer Genome Atlas (TCGA) colon adenocarcinoma (COAD) related to gene expression profiles and levels of DNA damage were downloaded from the data hubs of UCSC Xena database. The ChIP data and gene expression information of SIRT6 knockout, etoposide (VP16), and ionizing radiation (IR) treatment were downloaded from the Gene Expression Omnibus datasets (https://www.ncbi.nlm.nih.gov/geo/). The genes in DNA damage-related pathways were download from Molecular Signatures Database (MSigDB).

### Cell Culture and Transfection

HCT-116, DLD-1, LoVo, and SW480 cells were purchased from ATCC (CCL-247, CCL-221, CCL-229, and CCL-228) and cultured in RPMI 1640 or Dulbecco’s modified Eagle’s Medium (Gibco, catalogue numbers 22400089 and 11965092) supplemented with 10% fetal bovine serum (Biowest, catalogue number S1810) at 37°C in a humidified incubator with 5% CO_2_. To induce DNA damage, cells were treated with different doses and time of camptothecin (Sigma-Aldrich, C9911) or etoposide (VP16, Sigma-Aldrich, E1383). SiRNAs were transiently transfected using Lipofectamine 3000 following the manufacturer’s instructions. The sequences of siSIRT6 are shown in **Supplementary Table S1**.

### Gene Ontology Analysis

Gene Ontology (GO) analysis was performed using the enrichGO function of clusterProfiler package (version 3.18.1) (18). The *p*-value cutoff was set as 0.05, and the sub-ontology of biological processes was studied. The items with *P <*0.05 were considered significant enrichment on the genes with promoter occupied by histone crotonylation.

### Weighted Gene Co-Expression Network Analysis

Weighted gene co-expression network analysis (WGCNA) of the genes with promoter occupied by histone crotonylation in COAD samples was performed using the WGCNA package (version 1.67) (19). The result of cluster dendrogram was displayed using plotDendroAndColors function, and the module–trait relationship was displayed using the labeledHeatmap function. The DNA-damage-related complexes and abstract processes with absolute value of correlation coefficient greater than 0.2 and *p <*0.05 were considered to be related to the module. The functional enrichment of genes in every module was analyzed using GO analysis.

### Gene Set Enrichment Analysis

Gene Set Enrichment Analysis (GSEA) was performed using the fgseaLabel function of fgsea package (version 1.16.0) (20). Seven gene sets representative for histone crotonylation were used. The parameters of nperm, minSize, and maxSize were set as 10,000, 15, and 5,000, respectively. The COAD samples were separated into two groups according to the median value of DNA damage abstract or SIRT6 in paradigm IPLs. The rank of genes was based on the fold-change of gene expression between two sample groups, which was analyzed using the edgeR package (version 3.32.1) (21).

### Fisher’s Exact Test

Fisher’s exact test was applied to identify the relationship between two groups of genes (22). The test was performed using the fisher.test function of stats package of R (version 4.0.4) (23). The results with *P <*0.05 were considered to be significantly correlated and displayed using heatmap.2 function of gplots package (24). The white spaces meant that *P >*0.05.

### Pearson Correlation Analysis

The relationship between the expression of genes with promoter occupied by H3K27cr and the values of DNA-damage-related complexes and abstract processes in paradigm IPLs of COAD was determined using cor function. The parameter of the method was set as “pearson”. The result was displayed using ggcorrplot package (version 0.1.3) (25).

### Western Blotting

Total protein was extracted from cells using a cell extraction buffer [50 mM Tris-HCl (pH 8.0), 4 M urea, and 1% Triton X-100] containing a protease inhibitor cocktail (Roche Diagnostics, catalogue number 04693132001). The samples were subsequently resolved using SDS-PAGE and analyzed by western blotting. The following antibodies were used: anti-H3 (CTS, #4499, 1:1,000), anti-γH2AX (CTS, #9718, 1:1,000), anti-H3K27cr (RevMab Biosciences, 31-1287-00, 1:800), anti-SIRT6 (Proteintech, #13572-1-AP, 1:1,000), anti-ACTB (Proteintech, #20536-1-AP, 1:5,000), anti-rabbit IgG (KPL, 074-1506, 1:5,000), and anti-mouse IgG (KPL, 074-1806, 1:5,000). The intensity of image was measured by Image J2 software (https://imagej.net/Fiji/Downloads).

### Quantitative Real-Time Reverse Transcription PCR

Total RNA was isolated using RNA iso Plus (Takara, catalogue number D9108B) and reverse-transcribed using a ReverTra Ace qPCR RT Kit (TOYOBO, catalogue number FSQ-101). Q-PCR was carried out using SYBR Green (TOYOBO, catalogue number QPK-201). Each experiment was repeated at least three times. The expression level was normalized to that of ACTB, and the relative fold expression was quantified using the 2^-ΔΔCt^ method. The sequences of the primers used are shown in **Supplementary Table S2**. Data were plotted using GraphPad Prism 8.0. For all comparisons, statistical analysis was performed using unpaired, two-tailed Student’s *t*-test. *P <*0.05 was considered statistically significant.

## Results

### Identification of the Relationship Between Histone Crotonylation and DNA Damage in Colon Cancer

**Figure 1:**
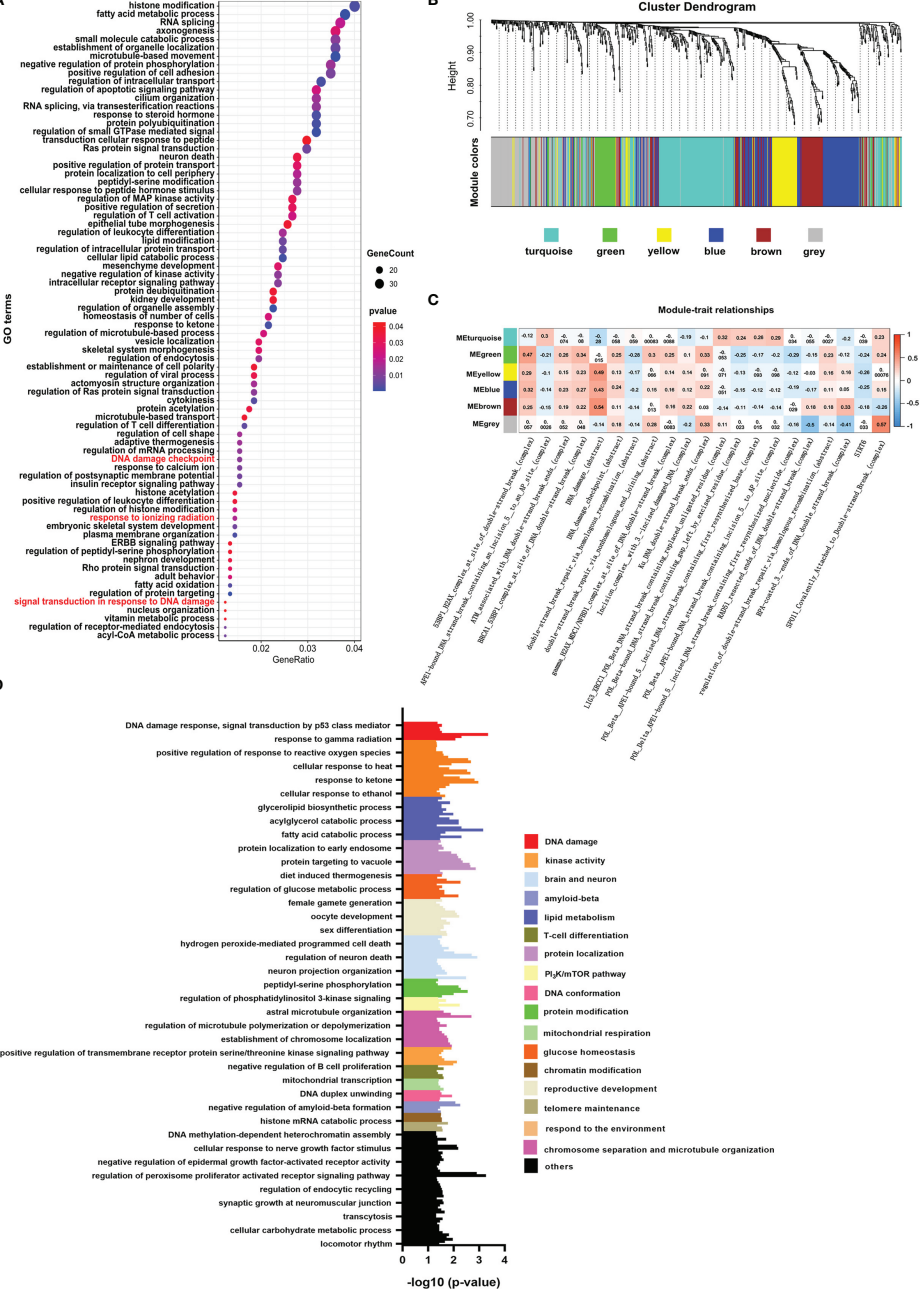
Histone crotonylation is related to DNA damage in colon cancer. **(A)** Gene ontology (GO) analyzing the enrichment items of genes with promoter occupied by histone crotonylation in sub-ontologies of biological processes (GSE124540). The circle size indicates the number of genes in each item. The color intensity indicates the negative base 10 logarithm of the *p*-value. The items with red color mean that the pathways were involved in DNA damage. **(B)** Weighted gene co-expression network analysis (WGCNA) of the gene co-expression module of the same gene set mentioned above in The Cancer Genome Atlas colon adenocarcinoma samples. **(C)** WGCNA of the relationship between each module and DNA-damage-related complexes or abstract processes in paradigm IPLs. The color intensity indicates the correlation coefficient. Red indicates a positive correlation, and blue indicates the opposite. **(D)** Gene Ontology analysis of the functional enrichment of genes enriched in the green module.

Like histone acetylation, the mark of histone crotonylation is strongly associated with the transcriptional activation of genes (26, 27). Whether it plays a role in DNA damage of colon cancer is not well studied. To identify the relationship between histone crotonylation and DNA damage, we exhibited GO analysis to study the functional pathway enrichment of genes with promoter occupied by histone crotonylation (GSE124540, ChIP-seq data of histone Kcr, crotonate plus). The result showed that genes with promoter occupied by histone crotonylation were associated with the DNA-damage-related pathway, including signal transduction in response to DNA damage, DNA damage checkpoint, and response to ionizing radiation (**Figure 1A** and **Supplementary Table S3**). To further confirm our idea, the same gene set was used to perform the WGCNA in the TCGA COAD samples. The gene co-expression module was calculated, and 5 functional modules were identified (**Figure 1B**). The level of DNA-damage-related complexes or abstract processes in the paradigm IPLs of colon cancer patients is used to infer the activity of DNA damage (17). Therefore, to identify the association between histone crotonylation and the activity of DNA damage, the relationship between each module and DNA-damage-related complexes or abstract processes in paradigm IPLs was analyzed. The DNA-damage-related complexes or abstract processes with an absolute value of correlation coefficient greater than 0.2 and *P <*0.05 were considered to be related to the module. In the green module, there were 15 correlation coefficients whose absolute value were greater than 0.2, suggesting that the green module was closely related to DNA damage (**Figure 1C**). Subsequently, the GO analysis was performed to study the functional pathway enrichment of genes in each module. The result revealed that some genes in the green module were involved in DNA-damage-related pathway (**Figure 1D**, **Supplementary Figures S1–S4**, and **Supplementary Table S4**), indicating that some genes with promoter occupied by histone crotonylation were associated with DNA damage in colon cancer.

**Figure 2:**
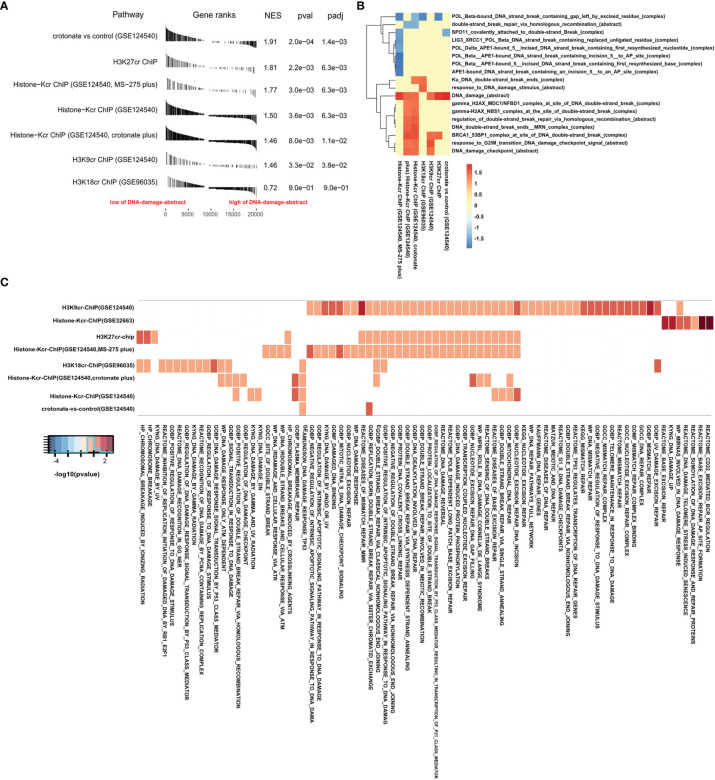
Gene Set Enrichment Analysis (GSEA) confirmed that histone crotonylation is related to DNA damage in colon cancer. **(A)** GSEA showing DNA damage abstract value evaluated in the context of 7 gene sets representative for histone crotonylation in colon adenocarcinoma samples. **(B)** Heat map showing the enrichment of genes with promoter occupied by histone crotonylation in each DNA-damage-related complex or abstract process in GSEA. The normalized enrichment score is shown as color intensity. Blue indicates DNA-damage-related complex or abstract process negatively relevant to histone crotonylation, and red indicates the opposite. **(C)** Heat map showing the genes with promoter occupied by histone crotonylation involved in multiple DNA-damage-related pathways in Fisher’s exact test (*P* < 0.05). The color intensity indicates the negative base 10 logarithm of the *p*-value. The white spaces meant that *P >*0.05.

The level of DNA damage abstract in paradigm IPLs can reflect the activity of DNA damage of colon cancer (17). To further study the connection between histone crotonylation and DNA damage in colon cancer patients, GSEA was applied to compare the value of DNA damage abstract of colon cancer patients to 7 gene sets related to histone crotonylation in previous studies (26, 28, 29). The COAD samples were separated into two groups according to the median value of DNA damage abstract, and then the enrichment of these 7 gene sets was analyzed. The result showed that, except for genes with promoter occupied by H3K18cr, the published histone-crotonylation-related gene sets were significantly enriched in DNA damage abstract (**Figure 2A** and **Supplementary Table S5**). We found that the gene set of “H3K9cr ChIP” was significantly enriched, which was consistent with the reduction of H3K9cr during DNA damage (14). Like the gene set of “H3K9cr ChIP”, the gene set of “H3K27cr ChIP” was also significantly enriched in patients with a low level of DNA damage abstract, suggesting that the level of H3K27cr might also decrease during DNA damage. Subsequently, the same analysis was performed to investigate the association between these 7 gene sets and other DNA-damage-related complexes or abstract processes of the paradigm IPLs in colon cancer patients. The COAD samples were divided into two groups according to the median value of each DNA-damage-related complex or abstract process. Then, the enrichment of these 7 gene sets was analyzed using GSEA. The enrichment with *P <*0.05 was displayed using a heat map. The result illustrated that these 7 gene sets markedly enriched on many DNA-damage-related complexes or abstract processes in the paradigm IPLs, suggesting that histone crotonylation was associated with DNA damage in colon cancer patients (**Figure 2B** and **Supplementary Table S6**). Fisher’s exact test was also employed to compare these 7 gene sets to the DNA-damage-related pathways in the Molecular Signatures Database (MSigDB). The result suggested that genes with promoter occupied by H3K9cr were enriched in multiple pathways (49 of the 78 DNA-damage-related pathways) (**Figure 2C** and **Supplementary Table S7**). Additionally, genes with promoter occupied by H3K27cr were also enriched in multiple pathways (27 of the 78 DNA-damage-related pathways).

### Identification of Association Between H3K27cr and DNA Damage in Colon Cancer

**Figure 3:**
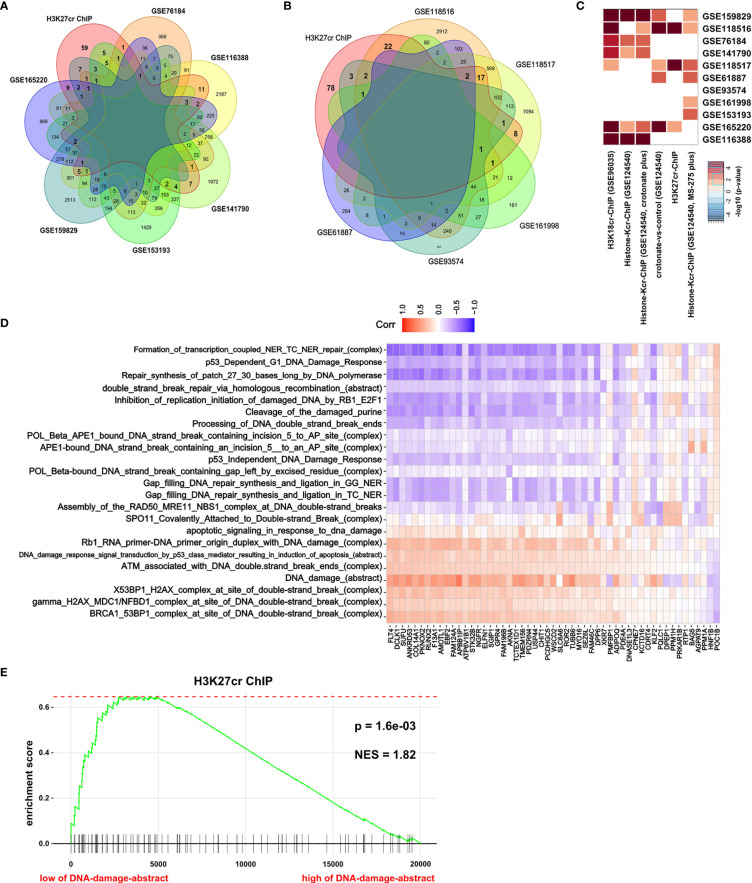
H3K27cr is related to DNA damage in colon cancer. **(A, B)** The overlay of genes with H3K27cr enrichment on their promoter regions (−5 kb, +1 kb) and genes with expression change (fold-change >2) during ionizing radiation (IR) **(A)** or VP16 **(B)** treatment. **(C)** Heat map showing the genes with promoter occupied by histone crotonylation involved in multiple gene sets induced by IR or VP16 treatment in Fisher’s exact test. The color intensity indicates the negative base 10 logarithm of the *p*-value. The white spaces meant that *P >*0.05. **(D)** Heat map showing the association between the expression of genes with H3K27cr enrichment on their promoter regions (−5 kb, +1 kb) and the value of DNA-damage-related complexes and abstract processes of paradigm-inferred pathway levels (IPLs) in Pearson correlation analysis. The color intensity indicates the value of the correlation coefficient. Blue indicates a negative correlation, and red indicates the opposite. **(E)** Gene Set Enrichment Analysis enrichment score curve showing that genes with promoter occupied by H3K27cr were enriched in colon cancer patients with a low level of DNA damage abstract in paradigm IPLs. The green curve indicates the enrichment score.

H3K27cr, a kind of histone crotonylation, has been found to accelerate gene transcription during spermatogenesis (26). In **Figure 2**, it is revealed that genes with promoter occupied by H3K27cr were associated with DNA damage in colon cancer. Ionizing radiation (IR) can directly disrupt atomic structures, leading to chemical and biological changes (30). Etoposide (VP16) is a drug that induces DNA double-strand breaks by poisoning topoisomerases II as covalent adduct (31). IR and VP16 are frequently utilized to induce DNA damage in living cells. Therefore, to further verify our conclusion, we compared the overlay between genes with promoter occupied by histone crotonylation and genes induced by IR or VP16 treatment. The result displayed that some genes induced by IR or VP16 treatment overlapped with some genes occupied by H3K27cr on the promoter (**Figures 3A, B**, **Supplementary Figures S4, S5**, and **Supplementary Tables S8, S9**). Additionally, Fisher’s exact test was also performed to study the association between genes with promoter occupied by histone crotonylation and genes induced by IR or VP16 treatment. The results with *P <*0.05 were displayed using a heat map. The result showed that genes with promoter occupied by H3K27cr were enriched in three gene sets induced by IR or VP16 treatment (**Figure 3C**). The association between the expression of genes with promoter occupied by H3K27cr and the levels of DNA-damage-related complexes or abstract processes in colon cancer patients was also analyzed using Pearson’s correlation analysis. The result discovered that more than half of these genes were negatively correlated with DNA-damage-related complexes or abstract processes, suggesting that the level of H3K27cr might decrease during DNA damage in colon cancer (**Figure 3D** and **Supplementary Table S10**). To further confirm the connection between genes with promoter occupied by H3K27cr and DNA damage, GSEA was performed. The COAD samples were separated into two groups according to the median value of the DNA damage abstract in paradigm IPLs, and then the enrichment of “H3K27cr ChIP” gene set was analyzed. The result revealed that genes with promoter occupied by H3K27cr were significantly enriched in patients with a low level of DNA damage abstract, indicating that H3K27cr might be negatively correlated with DNA damage (**Figure 3E**).

### The Level of H3K27cr Was Reduced Following DNA Damage

**Figure 4:**
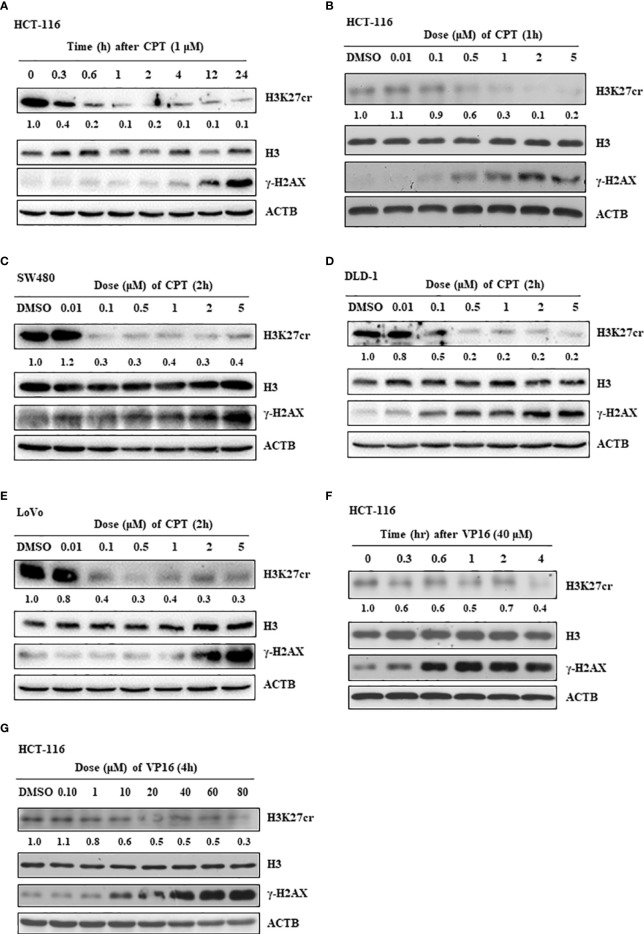
The level of H3K27cr is decreased during camptothecin (CPT) and VP16 treatment. **(A)** The western blot shows the level of H3K27cr in HCT-116 cells treated with CPT for different periods. **(B–E)** The western blot shows the level of H3K27cr in HCT-116 **(B)**, SW480 **(C)**, DLD-1 **(D)**, and LoVo **(E)** cells treated with CPT at different doses, respectively. **(F, G)** Immunoblotting analysis of the level of H3K27cr in HCT-116 cells treated with VP16 for different periods **(F)** and at different doses **(G)**. The ACTB acts as protein loading control. The DMSO acts as a negative control. The numbers under the H3K27cr panel represent the ratio of the level of H3K27cr *versus* H3 in the treatment group to that in the control group as analyzed by Image J2 software.

To further study our speculation, we determined the H3K27cr level in HCT-116 cells treated with CPT, a drug that induces DNA damage. The result revealed that H3K27cr was rapidly reduced in cells treated with CPT (**Figure 4A**). Notably, the reduction in H3K27cr level following DNA damage was also dependent on the dose of CPT treatment (**Figure 4B**), in the same way with the DLD-1, LoVo, and SW480 cells (**Figures 4C–E**). We also induced DNA damage using VP16 and found that the level of H3K27cr exhibited a reduction during VP16 treatment (**Figure 4F**). The reduction of H3K27cr depended on the dose of VP16 treatment as well (**Figure 4G**). In conclusion, we identified that the level of H3k27cr was reduced following DNA damage, consistent with the results described above using multiple bioinformatics.

### SIRT6 Regulated the Level of H3K27cr

**Figure 5:**
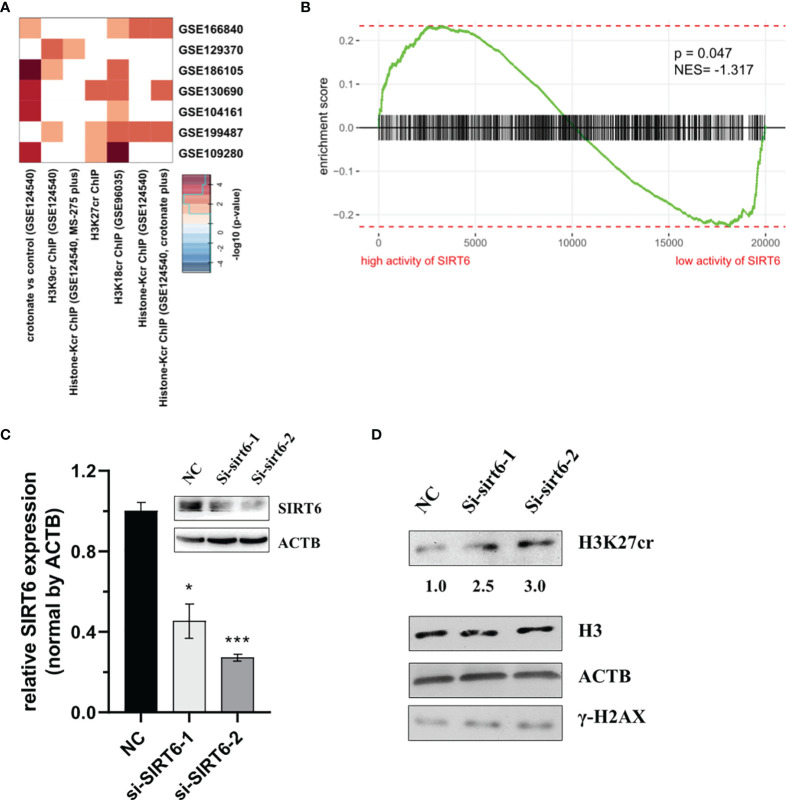
SIRT6 regulates the cellular level of H3K27cr. **(A)** Heat map showing that the genes with promoter occupied by histone crotonylation were involved in multiple gene sets regulated by SIRT6 in Fisher’s exact test. The color intensity indicates the negative base 10 logarithm of the *p*-value. The white spaces meant that *P >*0.05. **(B)** Gene Set Enrichment Analysis enrichment score curve showing that genes with promoter occupied by histone crotonylation (GSE124540) were enriched in colon cancer patients with a low activity of SIRT6. The green curve indicates the enrichment score. The negative enrichment score in the low activity end of SIRT6 means the upregulation of histone crotonylation pathway in the COAD samples with a low activity of SIRT6. **(C)** Western blot and qRT-PCR analysis of the level of SIRT6 in HCT-116 cells transiently transfected with siSIRT6 for 48 h. The level of mRNA expression was normalized to that of ACTB. Data are presented as means ± SD; *P < 0.05, ***P < 0.001; unpaired, two-tailed, Student’s *t*-test. **(D)** Immunoblotting analysis of H3K27cr level in HCT-116 cells transiently transfected with siSRIT6 for 48 h. The ACTB acts as protein loading control. The numbers under the H3K27cr panel represent the ratio of the level of H3K27cr *versus* H3 in the treatment group to that in the control group as analyzed by Image J2 software. NC, negative control (randomized siRNA sequences).

Previous studies have reported that the protein of sirtuin family accumulates at the DNA break sites. Among these, SIRT6 is responsible for a sensor of DNA double-strand break (32). Consequently, we hypothesized that SIRT6 might mediate the reduction of H3K27cr. To test this, the association between genes with promoter occupied by histone crotonylation and genes regulated by SIRT6 was studied using Fisher’s exact test. The results with *P <*0.05 were displayed using a heat map. The result showed that genes with promoter occupied by H3K27cr were enriched in three gene sets regulated by SIRT6 (**Figure 5A** and **Supplementary Table S11**). Additionally, the relationship between the activity of SIRT6 and genes with promoter occupied by histone crotonylation (GSE124540, ChIP-seq data of histone Kcr, and crotonate plus) was analyzed using GSEA. The COAD samples were separated into two groups according to the median value of SIRT6 in paradigm IPLs, and then the enrichment of “histone-Kcr ChIP (GSE124540, crotonate plus)” gene set was analyzed. It was found that genes with promoter occupied by histone crotonylation were significantly enriched in patients with a low activity of SIRT6 (**Figure 5B**, *P* = 0.047, normalized enrichment score = -1.32). We then transiently knocked down the expression of SIRT6 using siRNAs (**Figure 5C**) and found that the sirt6 knockdown resulted in a marked increase of H3K27cr level (**Figure 5D**), suggesting that SIRT6 might regulate the cellular level of H3K27cr.

## Discussion

DNA damage is considered to play an essential role in cancer initiation and development. The Kcr of total protein is found to be downregulated in liver, stomach, and kidney cancer and upregulated in thyroid, esophagus, colon, pancreas, and lung cancer (9). The level of histone crotonylation has been found to be abnormal in prostate cancer tissues. Additionally, previous studies suggested that the level of H3K9cr decreases in IR and VP16 treatment. However, no studies have linked histone crotonylation to DNA damage in colon cancer patients. In this study, we demonstrated that genes with promoter occupied by histone crotonylation were associated with the level of DNA damage in colon cancer patients using multiple bioinformatics analysis based on the colon cancer samples of the TCGA database. We found that the expression of genes with promoter occupied by H3K27cr and the level of DNA damage of colon cancer patients were negatively correlated. Additionally, we confirmed the reduction of H3K27cr during DNA damage at the cellular level.

Evidence show that histone post-translational modifications, including phosphorylation, methylation, acetylation, ubiquitination, neddylation, and poly(ADP-ribosyl)ation, are involved in the response to DNA damage (33)—for example, the phosphorylation of histone H2AX is a very critical step in DNA double-strand break repair, which can activate and recruit DNA repair factors (34). Besides this, H3K56ac is a key factor in stimulating the normal structural reassembly of chromatin after DNA double-strand break repair. Moreover, it is also a signal for the completion of the repair (35). It has been found that methylation, acetylation, and crotonylation can occur on lysine 27 of H3 (5). The modification of H3k27me3 has been found to accumulate at laser-induced DNA double-strand break sites (36). Additionally, the double-strand break hotspots are marked by H3K27ac modification (37). However, the relationship between H3K27cr and DNA damage in cancer is still unclear. In our study, we found that the expression of genes with promoter occupied by H3K27cr and the level of DNA damage in colon cancer patients have a negative correlation. Additionally, the level of H3K27cr was reduced in HCT-116 cells with CPT and VP16 treatment.

There is no report to prove the decrotonylase activity of SIRT6. In this study, we show that SIRT6 can decrease the cellular level of H3K27cr. An *in vitro* study using a chemical proteomics approach previously demonstrated that SIRT1-3 catalyzed the hydrolysis of lysine-crotonylated histone peptides (10). SIRT6 is a member of sirtuin family, which may also contain the activity of histone decrotonylases, although Xiucong Bao *et al.* claimed that little hydrolysis of the crotonyl peptide was observed in SIRT6 treatment. However, the protein of SIRT6 that they used was a truncated SIRT6 (1–314), which might affect its decrotonylase activity. Additionally, the fact that they performed the experiment *in vitro* instead of *in vivo* might also result in the disruption of the decrotonylase activity of SIRT6 during the purification of such. Moreover, they detected the H3K4cr peptide and found that the decrotonylase activity of SIRT6 to the H3K4cr peptide was weak, which cannot directly indicate that the decrotonylase activity of SIRT6 at the position of H3K27cr was also weak. In this study, we silenced the expression of SIRT6 in the living cells and detected the intercellular level of H3K27cr. Since our assay was different from that of Xiucong Bao, it was understandable that our results differed from theirs.

## Conclusion

In summary, we discovered the relationship between histone crotonylation and DNA damage in colon cancer. The level of H3K27cr was reduced during DNA damage in colon cancer, which might be mediated by SIRT6. Our findings connected SIRT6, histone crotonylation, DNA damage, and colon cancer, providing potential novel therapeutic targets. Further studies are needed to elucidate the biological functions of histone crotonylation and the underlying regulatory mechanisms as well as the balance between histone crotonylation and de-crotonylation during DNA damage of cancer.

## Data Availability Statement

The original contributions presented in the study are included in the article/**Supplementary Material**. Further inquiries can be directed to the corresponding authors.

## Author Contributions

ML designed and conducted the experiments, analyzed the data, and wrote and revised the manuscript. WC, XS, and WZ detected the level of H3K27cr in colon cancer cells with CPT and VP16 treatment. SG and DL detected the relationship between H3K27cr and SIRT6. DP designed the experiments and supervised the project. All authors contributed to the article and approved the submitted version.

## Funding

This work was supported by the Youth Program of National Natural Science Foundation of China (grant no. 31900540).

## Conflict of Interest

The authors declare that the research was conducted in the absence of any commercial or financial relationships that could be construed as a potential conflict of interest.

## Publisher’s Note

All claims expressed in this article are solely those of the authors and do not necessarily represent those of their affiliated organizations, or those of the publisher, the editors and the reviewers. Any product that may be evaluated in this article, or claim that may be made by its manufacturer, is not guaranteed or endorsed by the publisher.
