# Supplementary material for: Reduction of H3K27cr Modification During DNA Damage in Colon Cancer
Source: Front Oncol. 2022 Jul 22;12:924061. doi: 10.3389/fonc.2022.924061 (PMC9353715; doi:10.3389/fonc.2022.924061)
Supplement: Supplementary file 12 [file DataSheet_1.docx]

**Reduction of H3K27cr Modification During DNA Damage**

Meijian Liao^1*^, Weiwei Chu^2^, Xiaolin Sun^1^, Wendan Zheng^1^, Shoucui Gao^1^, Danhua Li^1^, Dongsheng Pei^1*^

^1^Department of Pathology, Xuzhou Medical University, Xuzhou 221004, P.R. China

^2^School of Pharmaceutical Sciences (Shenzhen), Sun Yat-sen University, Shenzhen, China

**Running title: SIRT6 Regulates H3K27cr Modification**

**Conflict of interest:** The authors declare no potential conflicts of interest

**Keywords:** Histone crotonylation; H3K27cr; DNA damage; SIRT6; Etoposide

***Correspondence:**

[liaomeijian@xzhmu.edu.cn](mailto:liaomeijian@xzhmu.edu.cn) (Meijian Liao)

[dspei@xzhmu.edu.cn](mailto:dspei@xzhmu.edu.cn) (Dongsheng Pei)

**
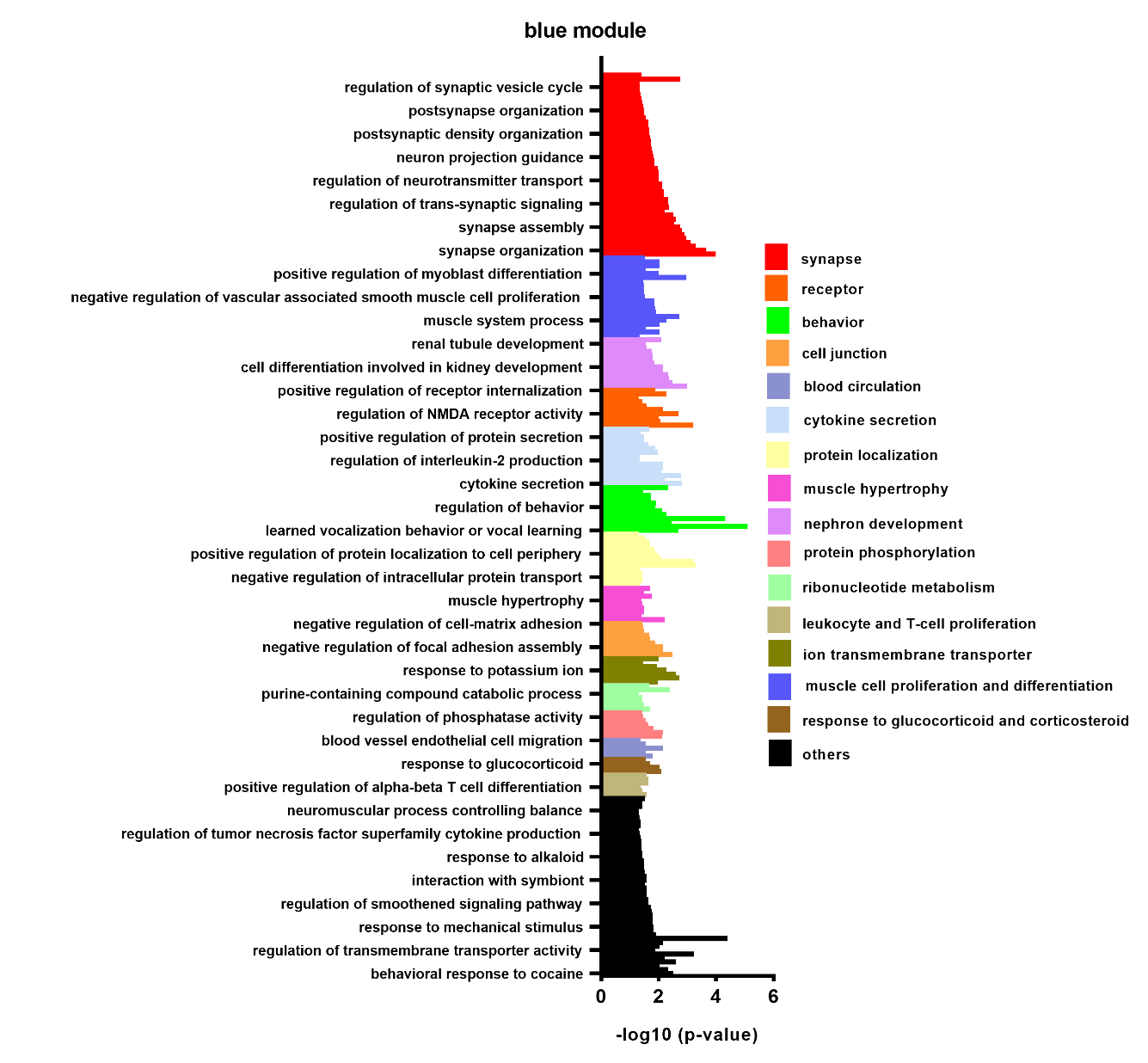
**

**Figure S1.** Gene ontology (GO) analysis functions of genes enrichment in blue module. Related to Figure 1.


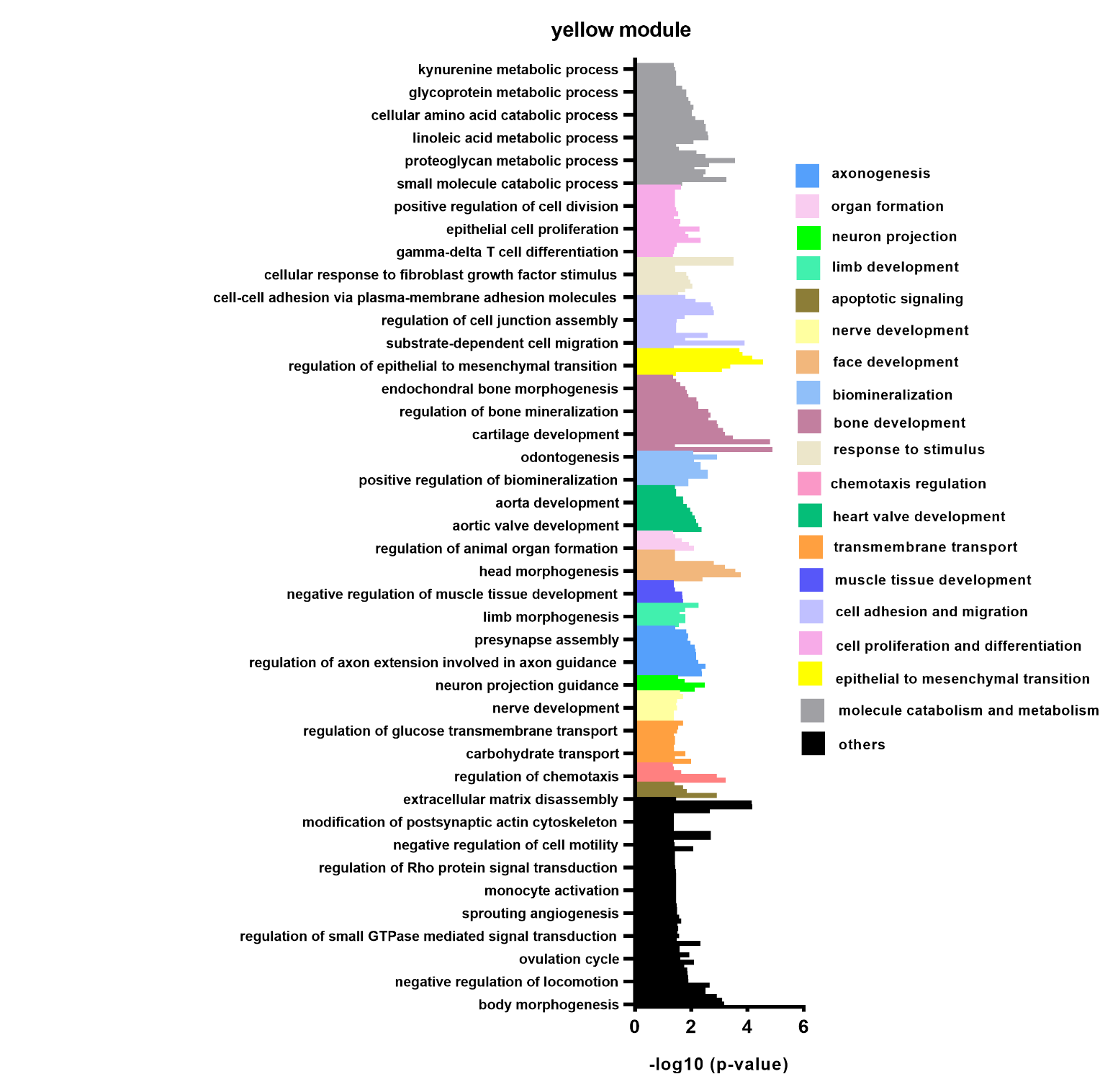


**Figure S2.** GO analysis functions of genes enrichment in yellow module. Related to Figure 1.


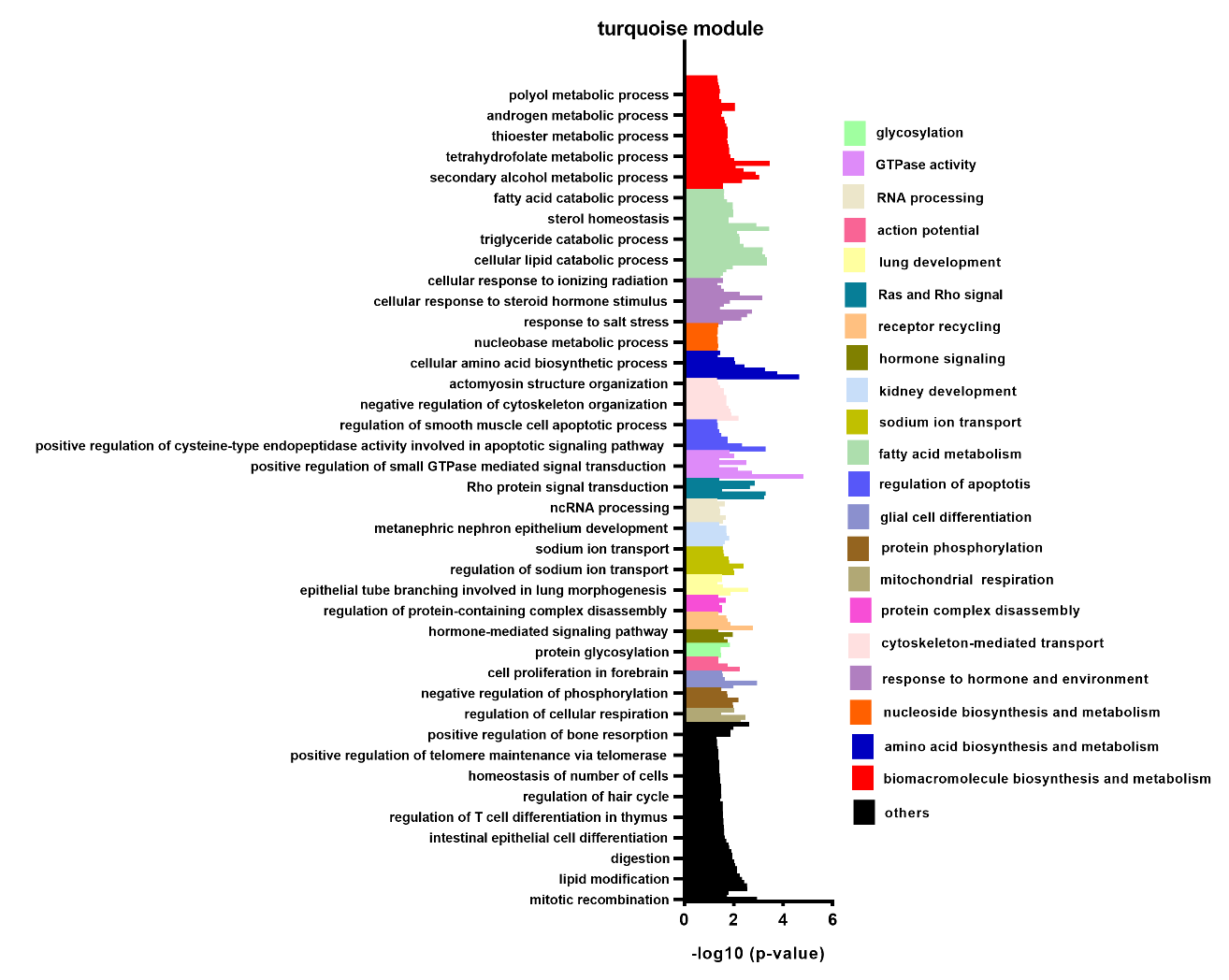


**Figure S3.** GO analysis functions of genes enrichment in turquoise module. Related to Figure 1.


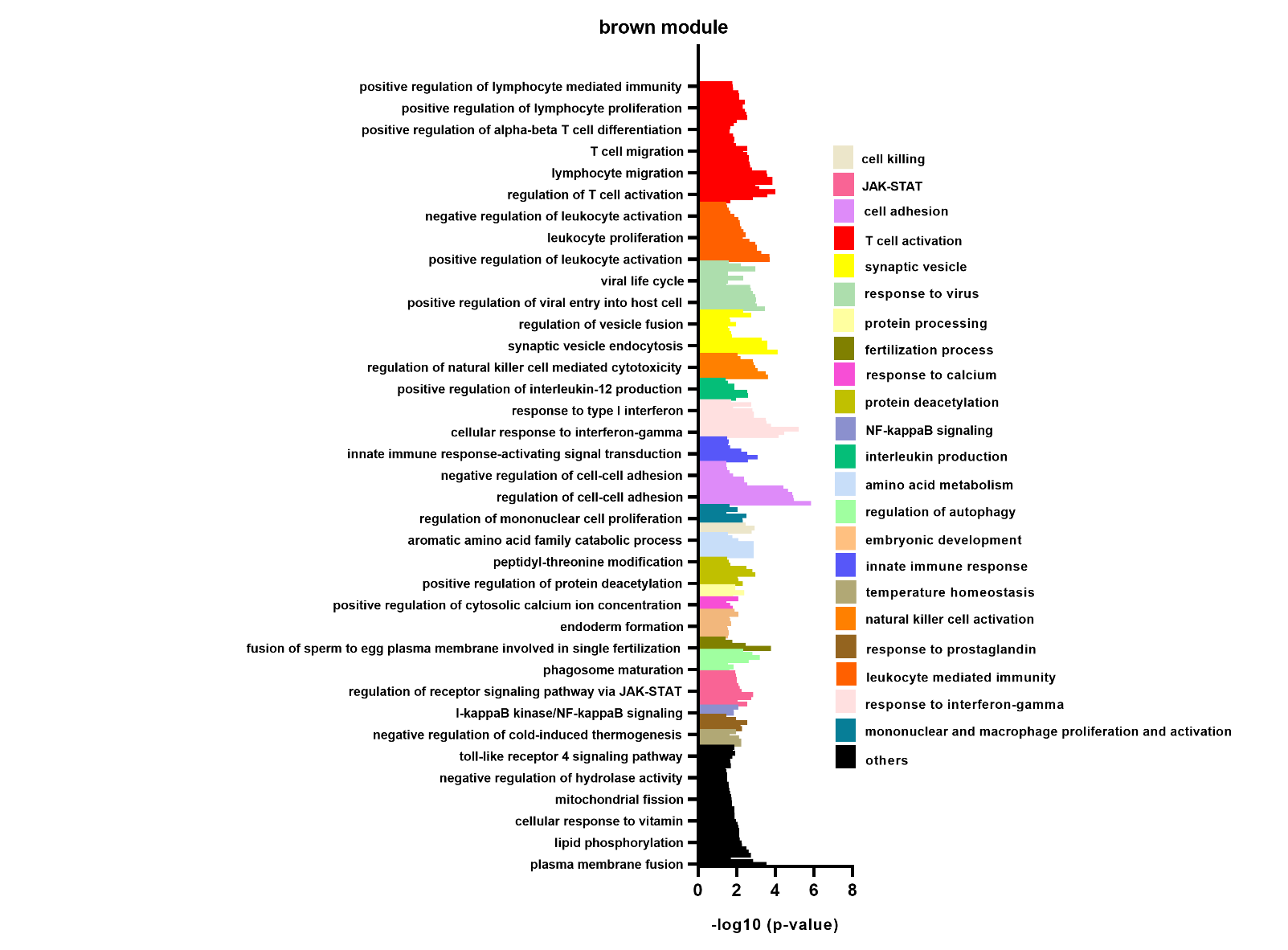


**Figure S4.** GO analysis functions of genes enrichment in brown module. Related to Figure 1.


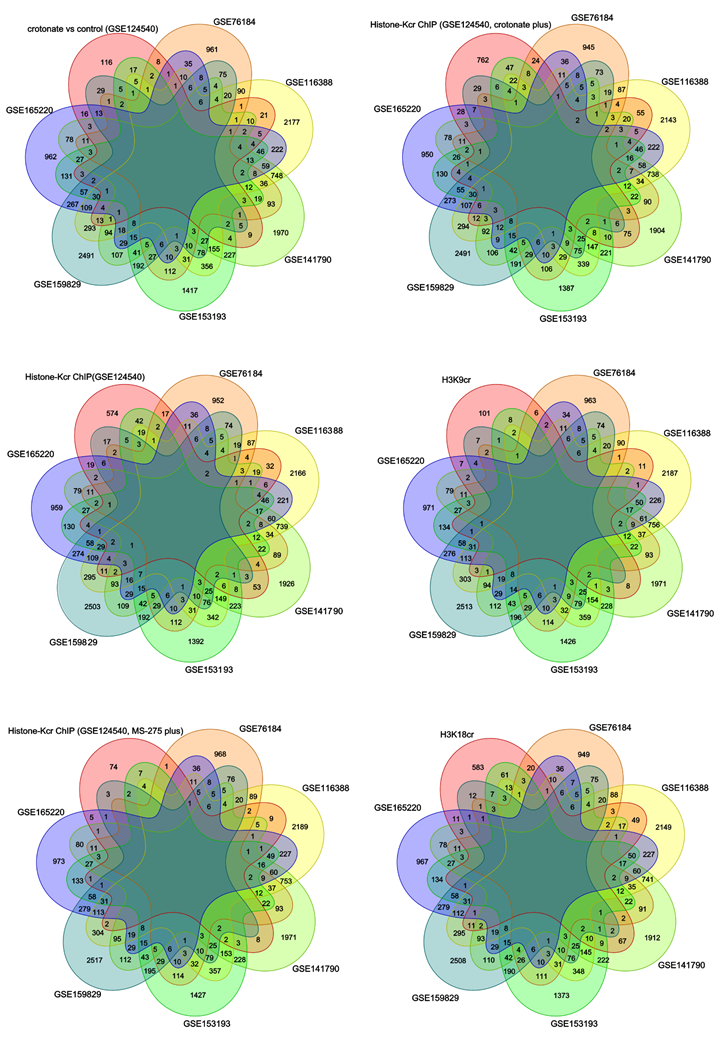


**Figure S5.** The overlay of genes with histone crotonylation enrichment on their promoter regions (−5 kb, +1 kb) and genes with expression change (fold-change >2) during IR treatment. Related to Figure 3.


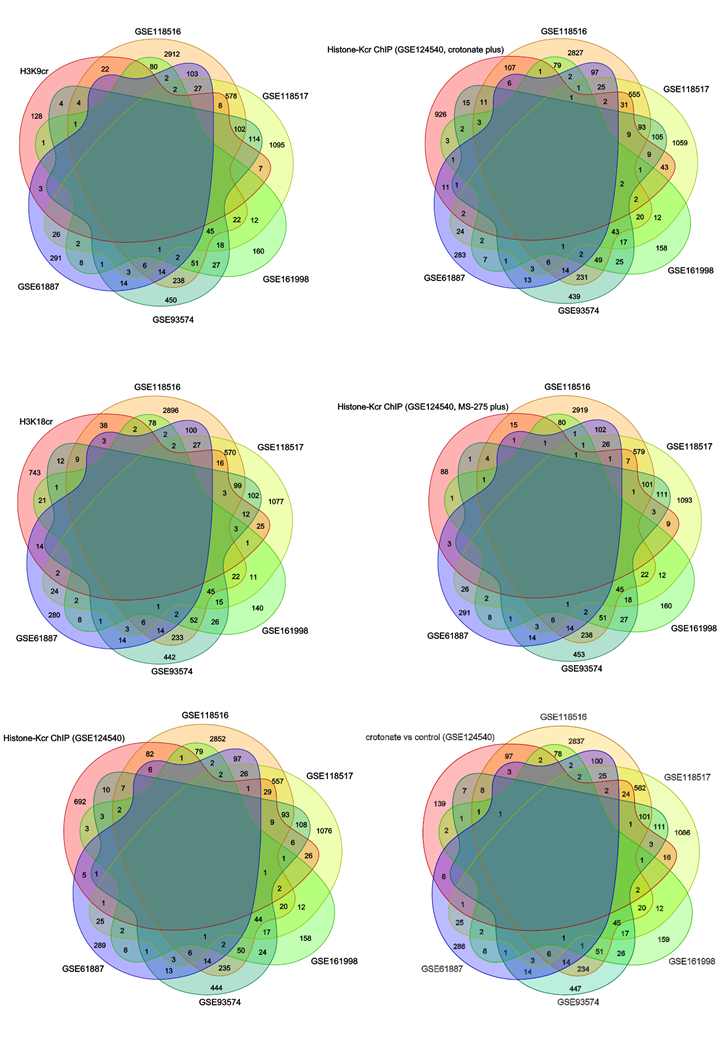


**Figure S6.** The overlay of genes with histone crotonylation enrichment on their promoter regions (−5 kb, +1 kb) and genes with expression change (fold-change >2) during VP16 treatment. Related to Figure 3.
